# Survival and Treatment Modalities in Primary Vaginal Melanoma—Case Report and a Narrative Review

**DOI:** 10.3390/jcm13133771

**Published:** 2024-06-27

**Authors:** Paweł Guzik, Martyna Łukasiewicz, Magdalena Harpula, Paweł Zając, Marcin Żmuda, Marcin Śniadecki, Paweł Topolewski

**Affiliations:** 1 Clinical Department of Gynecology and Obstetrics, City Hospital, 35-241 Rzeszów, Poland; harpula.magda@gmail.com (M.H.); zajacp71@gmail.com (P.Z.); 2 Medical University of Gdańsk, 17 Smoluchowskiego St., 80-241 Gdańsk, Poland; martyna.lukasiewicz@gumed.edu.pl (M.Ł.); ptopolewski@gumed.edu.pl (P.T.); 3 Pathology Department, Clinical Provincial Hospital no 2, 35-241 Rzeszów, Poland; martinuz@tlen.pl; 4 Department of Gynecology and Obstetrics, Medical University of Gdańsk, 17 Smoluchowskiego St., 80-241 Gdańsk, Poland; marcin.sniadecki@gumed.edu.pl

**Keywords:** melanoma, vaginal, adjuvant therapy, mucosal melanoma, primary vaginal melanoma, nivolumab, ipilimumab

## Abstract

**Background/Objectives:** Primary vaginal melanoma (PVM) is a rare cancer representing five percent of vaginal cancers and less than one percent of all female vaginal melanomas, with an incidence rate of 0.46 per million women per year. The aim of this study was to present a case of combined therapy and conservative surgical treatment in a young patient with PVM and to perform a systematic review of the same subject. **Methods**: We performed a narrative review of the literature and presented a case report. **Results**: The review yielded a total of 43 articles. We presented treatment modalities and survival outcomes. The presented case involved a combination of surgical treatment with adjuvant therapy comprising nivolumab and ipilimumab. **Conclusions**: PVM is a disease with a poor prognosis; however, new treatment options are promising and have a great chance of significantly improving survival. The combination of the wide local excision of the primary lesion followed by adjuvant therapies results in the best outcomes in the treatment of PVM. Future clinical studies are warranted to provide new evidence for the treatment outcomes of nonsurgical, metastatic PVM and the adjuvant treatment of PVM.

## 1. Introduction

Primary vaginal melanoma (PVM) is a rare cancer representing five percent of vaginal cancers and less than one percent of all female vaginal melanomas, with an incidence rate of 0.46 per million women per year [1]. The average age of incidence is 68 years, with a predominance in the white population. It mostly occurs in postmenopausal women, and its etiology is unclear [2]. Patients are usually referred with primary, postmenopausal vaginal bleeding, pain, palpable mass, abnormal vaginal secretion, vaginal lumps, difficulty in sexual intercourse, and itching [2,3]. The lesion is usually found in the lower one-third of the anterior wall of the vagina. Metastases appear rapidly (average 10 months), mainly to the lungs (75%) and liver (43.8%). The five-year survival rate is estimated to be 15% (5–25%) [4,5]. Currently, no standards of effective treatment for patients with PVM have been defined. The most common treatment is surgical excision followed by an adjuvant radiotherapy, chemotherapy, immunotherapy, or mixed targeted therapy [3]. Surgical treatment includes wide local excision or radical excision with the aim of achieving a negative margin. Performing an extended abdominal surgery with lymphadenectomy is controversial and its benefit is doubted. Few adjuvant chemotherapy regimens have been proved beneficial alone or in combination in clinical trials. One of the recent promising results reported is treatment with interferon alpha, which has become a standard of care for some groups of patients [6]. Numerous novel methods of treatment are being tested in clinical trials. The aim of this study is to present a case of the application of a combined therapy and conservative surgical treatment in a young patient with PVM, and to perform a systematic review of the surgical and adjuvant strategies for PVM treatment and survival.

## 2. Materials and Methods

### 2.1. Search Strategy

This systematic review was performed according to the PRISMA 2020 (Preferred Reporting Items for Systematic Review and Meta-Analyses) guidelines [7]. The research questions are presented in the Patients, Interventions, Comparison, Outcome, and Study Design (PICOS) criteria in Table 1. We searched MEDLINE via the PubMed and Web of Science databases. Additionally, Cochrane Reviews were checked for applicable studies. We used the following keywords: melanoma, skin cancer, skin carcinoma, mucosal cancer, mucosal carcinoma, vagina, and primary. The search terms were combined using the Boolean operators ‘AND’ and ‘OR’. No filters were used. The full search strategy for MEDLINE via PubMed was as follows: ((melan*) OR (skin cancer) OR (skin carcinoma)) AND (vagi*) AND (prima*). The full search strategy for the Web of Science was as follows: ((ALL = (vagi*)) AND ALL = (prima*)) AND ALL = (melan*). The citations were synthesized using EndNote 21. Duplicates were removed using EndNote 21. The references of systematic reviews of similar subjects were scanned and analyzed for relevant studies that were not found in our search. The study was not registered in publicly accessible database.

**Figure 1:**
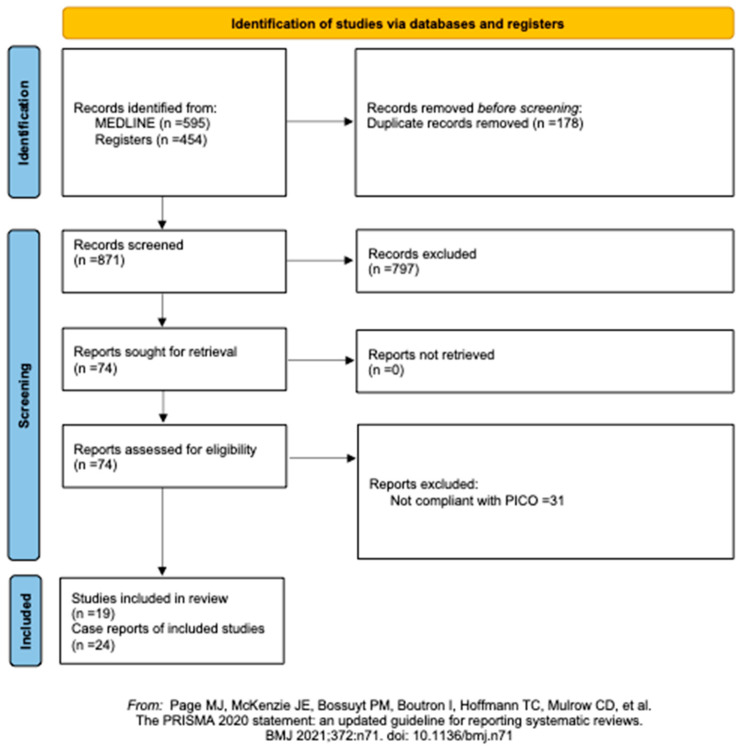
PRISMA flow chart of the literature search and article selection.

On 22 March 2024, the MEDLINE via PubMed and Web of Science databases were searched. The initial search yielded 1049 results. No relevant articles were found in the Cochrane Library. A total of 178 duplicates were identified by EndNote and removed. A total of 74 articles were chosen for full-text screening. Of these, 30 reports were excluded for not being compliant with the systematic review’s PICOS criteria. No studies were found or assessed for eligibility by reference analysis. A total of 46 reports met the inclusion criteria, including 24 case reports [2,6,8,9,10,11,12,13,14,15,16,17,18,19,20,21,22,23,24,25,26,27,28,29] and 19 cohort studies [1,30,31,32,33,34,35,36,37,38,39,40,41,42,43,44,45,46,47] (Figure 1).

### 2.2. Study Selection

The inclusion criteria were as follows: age > 18 years, histopathological diagnosis of primary vaginal melanoma, treatment modality, and survival.

The exclusion criteria were as follows: study outcomes not reported in English, not a full-text article type (conference meeting abstracts, posters, abstracts only, or no full-text available), disease other than primary vaginal melanoma reported in the study, and no survival outcomes. The selection process included reading titles and abstracts, reading full texts, and extracting data by two independent reviewers (P.T. and M.Ł.), with disagreements resolved by a third reviewer (P.G.).

### 2.3. Data Extraction

Two independent authors collected relevant data from the literature into spreadsheets for each included study, including the first author, year published, design, number of patients, age, depth of invasion, size, clinical stage at referral, treatment modality, radicality of surgery, number of adjuvant therapies and their modalities, recurrence rate, overall survival, and follow-up outcomes.

### 2.4. Risk of Bias and Literature Quality Evaluation

The quality of the included cohort studies was assessed by two independent researchers using the Newcastle–Ottawa scale [48], and disagreements were resolved by consulting a third researcher. The studies were assessed for selection (up to four stars), comparability (up to two stars), and exposure (up to four stars). The summary plots were created using the robvis tool [49].

## 3. Results

### 3.1. Case Report

A 29-year-old woman was referred to the hospital because of a suspected cervical polyp. The woman reported vaginal spotting and abdominal pain that had occurred for one month.

The examination carried out with the specula revealed a lofty pigmented lesion located in one-third of the proximal left vaginal sidewall, measuring two to three centimeters, with bleeding to the touch. The lesion was completely removed. Histopathological examination revealed malignant melanoma (nodular type) infiltrating the mucous membrane and deeper tissues. The lesion was removed without any margin of healthy tissue, with Breslow 15 mm and a Clark V level. Immunohistochemical staining for vimentin and melan A was positive. No metastatic foci were found in the imaging screening. The patient was referred to a reference center. After further imaging, metastases to the periaortic lymph nodes were found. No V600 BRAF mutation was detected by molecular examination. The patient was offered systemic treatment with possible future surgery in case of a reaction to the treatment. Due to the advanced stage of the neoplastic process, nivolumab and ipilimumab were included in the treatment. Imaging of the whole body three months after the end of treatment revealed numerous metastases in the uterus, brain, lungs, and liver. Palliative radiotherapy and chemotherapy were offered; however, no regression of the disease was achieved. One year after the diagnosis was made, the patient died of the disease.

### 3.2. Systematic Review

The cohort studies included were published between 1989 and 2023. A total of five case series and 14 retrospective studies were analyzed. The mean age ranged from 49 to 81.25 years. The primary treatment in most patients was surgery, which varied between wide local excision and extended surgery. The latter included vaginectomy with hysterectomy and bilateral salpingo-oophorectomy, with some cases extending to the pelvic exenteration. In two studies, external radiation was the first-line treatment. Surgical procedures were followed by systemic treatment in most cases. Adjuvant therapy was predominantly administered to patients undergoing local treatment. Among the adjuvant therapies, external radiotherapy was the most used. Doses of radiotherapy ranged from 45 Gy to 60 Gy, often delivered in fractions of 1.8 Gy to 2 Gy per session. Interferon alpha-2b was administered in 10 patients, but specific doses were not detailed. Chemotherapeutic agents were used in six patients and included dacarbazine (no dose specified) and temozolomide (administered in combination with radiotherapy). A total of 52 patients were treated with combined chemotherapy, interferon alpha-2b, or immunotherapy. There were no doses and schemes of immunotherapy specified. The recurrence rate varied significantly from 12.5% to 100%, although the size and heterogeneity of the groups were influential factors. Overall survival (OS) differed significantly between study groups and is not precisely estimable due to the use of median and mean values or the absence of reported data. Of the 828 patients across the studies, 514 patients have died of disease. Survival across the studies ranged from 2 months to 2325 months. Patients were recorded as deceased due to the poor prognosis of vaginal melanoma. Only four patients were reported to have no evidence of disease.

A summary of the cohort studies’ designs, cohort characteristics, interventions, and treatment outcomes are presented in Table 2.

The case reports included were published between 1989 and 2023. The ages ranged from 31 to 80 years, with a mean of 58 years. The depth of tumor invasion varied from 2 mm to 13 mm. The primary treatment for all patients was surgery; 12 patients underwent radical procedures (total vaginectomy and/or hysterectomy with or without abdominal exenteration), 7 patients underwent limited surgery (including wide local excision), and 3 patients underwent partial vaginectomy. In one instance, due to the advanced stage of the disease, the operation involved laparoscopic pelvic lymph node biopsy alone. One patient was lost to follow-up immediately after receiving the pathological examination results, resulting in no medical intervention being administered. Adjuvant therapy was administered in most patients and included external radiotherapy, brachytherapy, immunotherapy, and chemotherapy. Six patients underwent chemotherapy, eight patients underwent immunotherapy, and eight patients underwent radiotherapy (brachytherapy or external radiotherapy). Specific chemotherapy agents were not reported, with mentions of “chemotherapy” or “palliative chemotherapy”. Eight patients underwent radiotherapy, and one of them underwent brachytherapy. In some cases, adjuvant treatment modalities were combined. In eight patients, adjuvant therapy was not applied. The therapeutic success was gauged by the occurrence of recurrence, which was observed in nine patients. OS was only reported in four patients because the patients died before the follow-up visit. Twelve patients were alive and had no signs of disease during the last follow-up visit, four were lost to follow-up, four died from disease, and four were alive but had disease.

A summary of the patients included in the case reports, interventions, and treatment outcomes is presented in Table 3.

**Figure 2:**
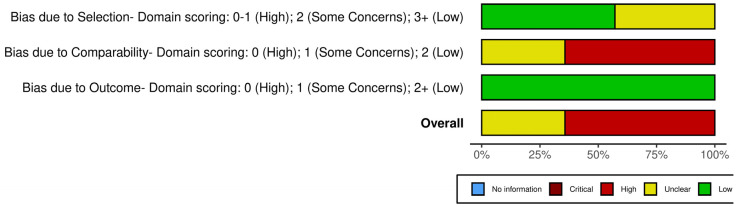
Summary plot of risk of bias assessment using the Newcastle–Ottawa scale.

The summary plot of the risk of bias assessment is presented in Figure 2. The studies were classified as having a moderate or high risk of bias due to concerns about comparability, possible selection bias that primarily arises from the rarity of the disease, and the selection of the study group (e.g., only patients with first-stage disease).

## 4. Discussion

This case is an illustration of the rapid and aggressive course of vaginal melanoma. PVM is a rare disease with no standardized treatment; therefore, the management of each patient with PVM is highly personalized and dependent on the stage of the disease. PVM diagnosis is often made in the late stage of the disease, with over 50% of patients having nodal involvement at the diagnosis [50,51]. Despite early diagnosis and aggressive treatment, the prognosis is poor. There is no optimal, efficient method for treating this type of cancer thus far. Currently, the basic method of treatment for vaginal melanoma is total surgical resection with a wide margin of healthy tissues [52]. Surgical resection is the only treatment modality for this disease for which there is evidence of complete cure. Surgery is the primary treatment modality in the absence of metastatic disease. Some authors have proposed extending the surgical procedure with the removal of regional lymph nodes by marking them with a sentinel node; however, this approach was proven not to be associated with improved survival [53,54]. In surgical resection, an 8 to 20 mm free margin should be obtained [46]. In some cases, that may be difficult due to a lesion location, and therefore the surgery can be extended. Although a meta-analysis was not performed due to the high heterogeneity of the results, we believe that our systematic review provides systematic evidence to the field in terms of (a) the range of surgeries that need to be performed and (b) the importance of adjuvant therapy that follows surgical treatment. The results of the present review show that wide local excision of the primary lesion followed by adjuvant therapies provides the greatest likelihood of a favorable disease course, although the prognosis remains poor. The results of the performed review suggest that performing extended surgeries with lymph node resection does not result in better overall survival. In our opinion, the greatest improvement to be made in PVM is in adjuvant therapies with a special focus on immunotherapy.

To date, limited studies have been carried out to assess adjuvant treatment after surgery. The use of radiotherapy as an adjuvant treatment may result in better local control of the disease; however, there is little evidence to support this statement [55]. Radiotherapy was one of the most frequent adjuvant treatment modalities in the literature review; however, it is suggested that radiotherapy may be used as an alternative for patients unfit for more aggressive treatment or as a synergy for other treatment modalities [55]. Wang et al. stressed the importance of chemotherapy and immunotherapy in combination with surgical confluence as the main therapeutic method for improving patient prognosis [56]. The use of interferon alpha was noted to prolong survival and is a promising treatment option; however, there are no high-quality studies assessing the effectiveness of such treatment in mucosal melanoma [21]. Targeted therapies are proven to be effective in treating cutaneous melanomas and most commonly include BRAF kinase inhibitors, often in combination with an MEK inhibitor. Despite mucosal melanomas rarely presenting BRAF or MEK mutations, they should be tested for these mutations, as the BRAF and MEK inhibitors may be considered as a treatment option. Mucosal melanomas sometimes have an activating mutation in the KIT, and sometimes tyrosine kinase inhibitors (imatinib and dasatinib) may be considered as an option [57]. Monoclonal antibodies against programmed cell death 1 (nivolumab) and against cytotoxic T-lymphocyte antigen 4 (ipilimumab) are in routine use in some countries. In the present case report, due to the inoperability of the lesion, the rapid appearance of metastases, and the lack of previous treatment, the patient was offered immunotherapy consisting of a combination of nivolumab and ipilimumab. Nivolumab has been proven to be an effective treatment option for skin melanoma [58]; however, there is no evidence supporting its use for treating mucosal melanoma. In a study by Robert et al., patients treated with nivolumab exhibited a longer survival time and inhibition of disease progression than patients treated with dacarbazine [59]. The use of ipilimumab in the monotherapy of mucosal melanoma prolonged the survival period without disease progression [60,61]. In October 2015, the use of ipilimumab–nivolumab in a combination therapy regimen for nonsurgical or metastatic melanoma in patients without the BRAF V600 mutation was registered in the United States of America, and in January 2016, it was used for all previously untreated patients with advanced disease. Previous studies have shown that nivolumab and ipilimumab treatment regimens and nivolumab monotherapy are superior to ipilimumab monotherapy in terms of progression-free survival time without disease and the percentage of objective responses [62,63]. In May 2016, the use of a combination of nivolumab and ipilimumab was approved in the European Union for patients with nonsurgical or metastatic melanoma, regardless of the BRAF mutation status. It is the first and only approved combination of immunocompetent molecules in the European Union that is currently undergoing clinical trials (NCT02626962, NCT04655157, and NCT01844505). Currently, the combination of ipilimumab with nivolumab may improve the response rate, resulting in a median progression-free survival of 11.5 months (2.9 months of progression-free survival for ipilimumab alone or 6.9 months for nivolumab alone) in metastatic disease [62]. However, when it comes to combined immunotherapy, the high rates of grade three and grade four toxicity should be taken under consideration. The clinical decision making is also complicated, with a modest improvement in the 3-year survival of 58% for patients treated with nivolumab–ipilimumab compared with 52% for patients with nivolumab monotherapy [64].

The rarity of the disease ensures that large-scale trials are unlikely to be feasible. Surgical excision remains the first line of treatment. In metastatic disease, immunotherapy, sometimes combined with chemotherapy or radiotherapy, has the greatest chance of prolonging the survival with the disease. Clinical decision making should be highly personalized and optimized based on the tumor biology, toxicity of the treatment, and tumor response. Including patients with PVM in the ongoing trials for new lines of treatment allows us to bring new evidence to the field and to offer the potential for a better survival for these groups of patients.

## 5. Conclusions

PVM is a disease with a poor prognosis; however, new treatment options are promising and have a great chance of significantly improving survival. The combination of the wide local excision of the primary lesion followed by adjuvant therapies still results in the best outcomes in the treatment of PVM. Future clinical studies are warranted to provide new evidence for the treatment outcomes of nonsurgical, metastatic PVM and the adjuvant treatment of PVM.

## Figures and Tables

**Table 1:** PICOS criteria used in the study.

**Patients**	Patients with histopathological confirmation of PVM ^1^.
**Interventions**	Treatment with intention to treat regardless of its type.
**Comparison**	Other modality of treatment or no treatment.
**Outcome**	Overall survival, recurrence-free survival, follow-up outcomes.
**Study Design**	Any study design.

^1^ Primary vaginal melanoma.

**Table 2:** Summary of study designs, cohort characteristics, interventions, and treatment outcomes.

First Author	Year Published	Design	Number of Patients	Age (Years)	Primary Treatment (Number)	Adjuvant Therapy	Recurrence Rate (%) OS (Months)	Follow-Up
Levitan Z et al.	1989	Case series	4	68.25 (mean)	WLE (3), none (1)	ER (1)	2 (50.0%) 103 mo, 2 mo, 5 mo, NR (1)	3 DOD
Borazjani G et al.	1990	Retrospective	10	69.5 (mean)	Anterior exenteration (2), radical vulvectomy and partial vaginectomy (1), TAH-BSO (1), WLE (1), none (5)	ChTx (3), ER (7)	NR 14.9 mo	8 DOD, 1 NED, 1 DOC
Khoo U S et al.	1991	Retrospective	4	64.5 (mean)	WLE (3), ER (1)	ER (3), ChTx (1)	2 (50%) 16 mo., 2 mo., NR (2)	2 DOD, 2 NED
Van Nostrand K. M. et al.	1994	Case series	8	64 (mean)	WLE (4), brachytherapy (2), total pelvic exenteration (1)	ER (2), none (5)	1 (12.5%) 10 mo (mean WLE), 44 mo (mean ES), 23.25 (mean all)	7 DOD, 1 NED
Irvin W et al.	1998	Retrospective	7	67.71 (mean)	WLE (4), brachytherapy (2), none (1)	ER (2), none (5)	6 (85.7%) 21.3 mo	6 DOD, 1 DOC
Gupta D et al.	2002	Retrospective	26	60 (mean)	WLE (10), anterior exenteration (7), hysterectomy with vaginectomy (3), vaginectomy (1), ER (2), ER + ChTx (1), NR (2)	ER (7), ChTx (3), ER + ChTx (7)	5 (19.2%) 18.4 mo	15 DOD
Gupta D et al.	2003	Case series	3	49 (mean)	WLE (1), ER (2)	ChTx (1)	1 (33.3%) 48 mo, 3 mo, 14 mo	1 DOD
Miner TJ et al.	2003	Retrospective	35	62 (median)	WLE (10), vaginectomy (2), ER (11), TAH-BSO (10), exenteration (2)	ER (16), interferon a-2b (7), vaccine (7)	18 (51.4%) NR	NR
Frumovitz M et al.	2010	Retrospective	37	60.6 (median)	WLE (28), pelvic exenteration (4), ER, ChTx or mixed (5)	ER (20)	33 (89.2%) 19.1 mo	2 DOD, 1 DOC, 34 NR
Huang Q et al.	2013	Retrospective	31	58.0 (median)	ES (11), WLE (11), ChTx (7), none (2)	Immunotherapy (19)	11 (35.5%) 20.1 mo	23 DOD
Xia L et al.	2013	Retrospective	44	56.7 (median)	WLE (21), RE (20), ChTx (3)	ER, ChTx (24)	30 (68.2%) 39.5 mo	21 DOD
Tasaka R et al.	2016	Retrospective	5	78.0 (median)	WLE (5)	Interferon-B (3), DAVFeron therapy (1)	5 (100.0%) 13.8 mo	4 DOD
Sinasac S et al.	2019	Retrospective	18	67.0 (mean)	WLE (10), no surgery performed (3), NR (5)	IFN-α (2)	14 (77.8%) 10.5 mo	11 DOD
Shakeel O et al.	2020	Retrospective	9	53.1 (mean)	WLE (4), TAH-BSO (2), none (3)	ER (8), ER + ChTx (1)	5 (55.6%) 25 mo	13 DOD
Wohlmuth C et al.	2020	Retrospective	463	69.5 (mean)	WLE (109), RE (77), DS (3) NS (41), no surgery performed (218) NR (15)	0	NR 16 mo	320 DOD, 143 NR
Khayyat A et al.	2022	Case series	4	81.25 (mean)	Palliative ER (2), ER (1), ChTx (1)	0	4 (100.0%) NR	3 DOD, 1 NR
Tian H et al.	2022	Retrospective	62	NR	Nonradical resection (29), radical resection (33)	ChTx, interferon-α2β, immunotherapy (49)	64 39.5 mo (mean ES), 25.4 mo (mean WLE)	38 DOD or lost to FU
Yazdanfard N et al.	2022	Retrospective	52	73 (mean)	WLE (34) ES (4) ER (3), ER +IT (3), ER + ChTx (1), NR (7)	ER (3), ER + immunotherapy (3), ER + electrochemotherapy (1)	33 (63.5%) 17.5 mo (median)	NR
Ishiguro A et al.	2023	Case series	6	67.5 (mean)	Brachytherapy (5), WLE (1)	ER (5), ER + immunotherapy (1)	4 (66.7%) NR	NR

Abbreviations: WLE—wide local excision, ER—external radiotherapy, mo—month, DOD—dead of disease, TAH-BSO—total abdominal hysterectomy with bilateral salpingo-oophorectomy, ChTx—chemotherapy, NR—not reported, NED—no evidence of disease, DOC—dead of other causes, NR—not reported, RE—radical excision, FU—follow-up, DS—debulking surgery, NS—not specified.

**Table 3:** Summary of patients in case reports, interventions, and treatment outcomes.

First Author	Year Published	Age (Years)	Depth of Invasion (mm)	Primary Treatment	Adjuvant Therapy	Recurrence (Yes/No) OS (Months)	Follow-Up
Schwartz J et al.	1989	79	NR	Local excision	ChTx	yes 37 months	DOD
Moros M L et al.	2004	40	13 mm	Radical hysterectomy and total vaginectomy	Immunotherapy, ER	yes 14 weeks	DOD
Gökaslan H et al.	2005	56	2 mm	Wide local excision	ChTx, immunotherapy	yes 16 months	DOD
Grenader T et al.	2008	31	NR	Posterior pelvic exenteration	ChTx	NR NR	Lost to FU
Albareda J et al.	2011	63	9 mm	Local excision	Immunotherapy, palliative ChTx	yes NR	Alive but with disease at 1-year FU
Kühn F et al.	2012	44	>3 mm	Multivisceral resection	ChTx, immunotherapy	yes 4 months	DOD
Androutsopoulos G et al.	2013	80	5 mm	Wide local excision	None specified	no NR	Alive at 5 months FU
Chaudhuri S et al.	2013	60	NR	Wide local excision	ER, ChTx	no NR	Alive at 1-year FU
Chen L F et al.	2014	35	NR	Radical surgery	Radiotherapy and immunotherapy	yes NR	Lost to FU
Rema P et al.	2016	NR	NR	Total pelvic exenteration	ER	no NR	Alive at 1-year FU
Chitrathara K et al.	2017	38	10 mm	Total vaginectomy and urethrectomy	ER	no NR	Alive at 14 months FU
Kalampokas E et al.	2017	75	4.85 mm	Partial vaginectomy and lymphadenectomy	None	no NR	Alive at 1-year FU
Lee C K et al.	2017	62	NR	Wide local excision	None	no NR	Alive at 6 months FU
Rapi V et al.	2017	72	NR	Wide local excision and lymphadenectomy	None	no NR	Alive at 5 months FU
Ahn, H Y et al.	2018	80	NR	Wide local excision and lymphadenectomy	None	no NR	Alive at 8 months FU
Zabroug S et al.	2018	31	4 mm	Partial colpectomy and lymphadenectomy	ER, brachytherapy	no NR	Alive at 1-year FU
Puri S et al.	2019	65	NR	Biopsy	ER	no NR	Alive at 6 months FU
Paravathaneni M et al.	2020	56	NR	Surgical excision	None	NR NR	Lost to FU
Tokumitsu R et al.	2020	56	2.5 mm	Total vaginectomy, lymphadenectomy, modified radical hysterectomy, bilateral salpingo-oophorectomy	None	no NR	Alive at 15 months FU
Guo N et al.	2021	58	NR	Local excision, lymph node dissection	Immunotherapy	no NR	Alive at 6 months FU
Yin P et al.	2022	55	NR	Radical vaginectomy and bilateral salpingo-oophorectomy	Immunotherapy, ER	yes NR	Alive at 1-year FU
Bai S et al.	2023	56	13 mm	Wide local excision and sentinel node biopsy	ChTx	no NR	Alive at last FU
Sticca G et al.	2023	68	11.3 mm	Posterior exenteration	None	yes NR	Lost to FU
Van Trappen P et al.	2023	73	8 mm	Robotic total vaginectomy and hysterectomy with lymphadenectomy	None	no NR	Alive at 4 months after surgery

Abbreviations: NR—not reported, ChTx—chemotherapy, DOD—dead of disease, ER—external radiation, FU—follow-up, OS—overall survival.

## Data Availability

No new data were created or analyzed in this study. Data sharing is not applicable to this article.
